# Metabolic Profiling of Plasma in Patients with Irritable Bowel Syndrome after a 4-Week Starch- and Sucrose-Reduced Diet

**DOI:** 10.3390/metabo11070440

**Published:** 2021-07-04

**Authors:** Hans Stenlund, Clara Nilholm, Elin Chorell, Bodil Roth, Mauro D’Amato, Bodil Ohlsson

**Affiliations:** 1 Umeå Plant Science Centre (UPSC), Department of Plant Physiology, Umeå University, 90187 Umeå, Sweden; hans.stenlund01@umu.se; 2 Department of Clinical Sciences, Lund University, 22100 Lund, Sweden; clara.nilholm@med.lu.se (C.N.); bodil.roth@med.lu.se (B.R.); 3 Department of Internal Medicine, Skåne University Hospital, 20502 Malmö, Sweden; 4 Department of Public Health and Clinical Medicine, Umeå University, 90187 Umeå, Sweden; elin.chorell@umu.se; 5 Gastrointestinal Genetics Laboratory, CIC bioGUNE-BRTA, 48160 Derio, Spain; mdamato@cicbiogune.es; 6 Ikerbasque, Basque Foundation for Science, 48009 Bilbao, Spain

**Keywords:** metabolomics, metabolic profiling, dietary advice, IBS, starch, sucrose

## Abstract

A 4-week dietary intervention with a starch- and sucrose-restricted diet (SSRD) was conducted in patients with irritable bowel syndrome (IBS) to examine the metabolic profile in relation to nutrient intake and gastrointestinal symptoms. IBS patients were randomized to SSRD intervention (*n* = 69) or control continuing with their ordinary food habits (*n* = 22). Food intake was registered and the questionnaires IBS-symptoms severity scale (IBS-SSS) and visual analog scale for IBS (VAS-IBS) were completed. Metabolomics untargeted analysis was performed by gas chromatography mass spectrometry (GC-MS) and liquid chromatography mass spectrometry (LC-MS) in positive and negative ionization modes. SSRD led to marked changes in circulating metabolite concentrations at the group level, most prominent for reduced starch intake and increased polyunsaturated fat, with small changes in the control group. On an individual level, the correlations were weak. The marked reduction in gastrointestinal symptoms did not correlate with the metabolic changes. SSRD was observed by clear metabolic effects mainly related to linoleic acid metabolism, fatty acid biosynthesis, and beta-oxidation.

## 1. Introduction

Irritable bowel syndrome (IBS) is defined as abdominal pain in combination with altered bowel habits, without any organic explanation [1]. Huge efforts are exerted to identify the pathophysiology behind the disease. One of the most important treatments is dietary advice with less intake of fermentable carbohydrates [2,3]. In the last years, several studies have found altered metabolite profiles in IBS patients in feces [4,5,6] or serum [7], which correlated with the severity of symptoms and visceral hypersensitivity [8,9,10]. Further, metabolite signatures (metabolomics) have been associated with several central sensitivity pain syndromes, including IBS [11]. 

Diets that have higher proportions of a specific macronutrient, e.g., fat, have an increased ability to oxidize the macronutrient primarily consumed [12,13,14]. In addition, the endogenous substrate concentrations increase after acclimating to high fat or high carbohydrate diet [14,15,16]. A high carbohydrate diet increases the muscle glycogen content and carbohydrate oxidation, with reduced release of fatty acids from intramuscular triacylglycerides (IMTG) and reduced-fat oxidation, whereas a high-fat diet increases IMTG and decreases carbohydrate oxidation [17]. Dietary intervention in IBS with yogurt resulted in normalized metabolites of the one-carbon metabolism pathways [18]. However, most dietary interventional studies in IBS focus on the effects of diet on the gut microbiome, and not much is known about the impact on the metabolite signature [19].

We conducted a 4-week dietary intervention with a starch- and sucrose-restricted diet (SSRD), previously shown to lower the irritable bowel syndrome-symptom severity scale (IBS-SSS) scores and to alleviate the gastrointestinal (GI) symptoms for IBS patients [3,20,21]. The primary aim of the present study was to examine the metabolic profile in relation to the SSRD dietary intervention, linking circulating plasma metabolites with the SSRD dietary advice in IBS patients on the average group level. No strict rules for food intake were given, which meant that the source and amount of food intake differed between participants, whilst following the Basic Dietary Guide for People with congenital sucrase-isomaltase deficiency (CSID) [22]. The secondary aim was to examine the relationship between metabolic profile, daily nutrient intake (AIVO calculator) [23], irritable bowel syndrome- symptom severity scale (IBS-SSS) [24], and visual analog scale for irritable bowel syndrome (VAS-IBS) scores [25], linking circulating plasma metabolites with macronutrients and GI symptoms in IBS patients on the individual level.

## 2. Results

### 2.1. Subject Characteristics

A total of 91 IBS patients were included in the study, with 22 IBS patients in the control group and 69 IBS patients in the intervention group (Table 1). Mean age was 47 ± 13 years (range 26–70 years) and body mass index (BMI) was 25.7 ± 4.4 kg/m^2^ (range 19.2–39.8 kg/m^2^). Age (*p* = 0.058), BMI (*p* = 0.471), and sex (*p* = 0.381) were equally distributed between groups at baseline, and no differences in IBS disease duration were observed (*p* = 0.523). The total symptom burden assessed as total IBS-SSS was equal in the intervention group (301.1 ± 73.5 (169–470)) and control group (302.1 ± 62.59 (176–393)) at baseline (*p* = 0.721), but markedly reduced in the intervention group (168.5 ± 104.4 (11-423)) compared to the control group (288.6 ± 78.14 (143–438)) after 4 weeks (*p* < 0.001). There were no differences in GI symptoms when accounting for the level of education (university degree or lower) or level of physical activity (data not shown), or use of probiotics (one in each group).

### 2.2. Clinical and Nutritional Effects of the SSRD Diet

The SSRD intervention led to a clear reduction in BMI (*p* = 0.002) for the intervention group compared with the control group, for the paired data (Table 1). For the dietary intake (AIVO) data, significant reductions in the intake of carbohydrates (*p* = 0.002), starch (*p* = 0.002), disaccharides (*p* = 0.014), and sucrose (*p* = 0.040) were observed, for the paired data. Noticeably, both the intervention group as well as the control group indicated food intake changes during the study period, by comparing the average baseline data with the average 4-week data. Moreover, it was obvious that the dietary intake was not strictly regulated and monitored in detail as the mean changes were not in line with the paired data, indicating great individual variations for both the intervention group as well as for the control group. As an example, monosaccharides were indicated to be reduced for the independent 4-week follow-up data while increased for the paired data. The average increment was 1.5 ± 88.5% for the control group and 29.5 ± 152.6% for the intervention group, indicating great variability between individuals and diet intake. In addition, reduced levels in monosaccharide intake were observed for 59.4% (41 out of 69) in the intervention group and 59.1% (13 out of 22) in the control group. Due to the large variability, monosaccharides showed no significant differences between the groups. Despite this, the proportional change in reported daily intake over the 4-week study period was greater on average for the intervention group. For the IBS symptoms questionnaires (IBS-SSS and VAS-IBS), significant improvements in health were observed for the intervention group. The total IBS-SSS scores were significantly changed for both the independent data (*p* < 0.001) and the paired data (*p* < 0.001). Furthermore, significant reductions in symptoms related to abdominal pain (*p* < 0.001), bloating and flatulence (*p* < 0.001), intestinal symptoms influence on daily life (*p* < 0.001), and diarrhea (*p* = 0.032) were observed, for the paired data. 

**Figure 1:**
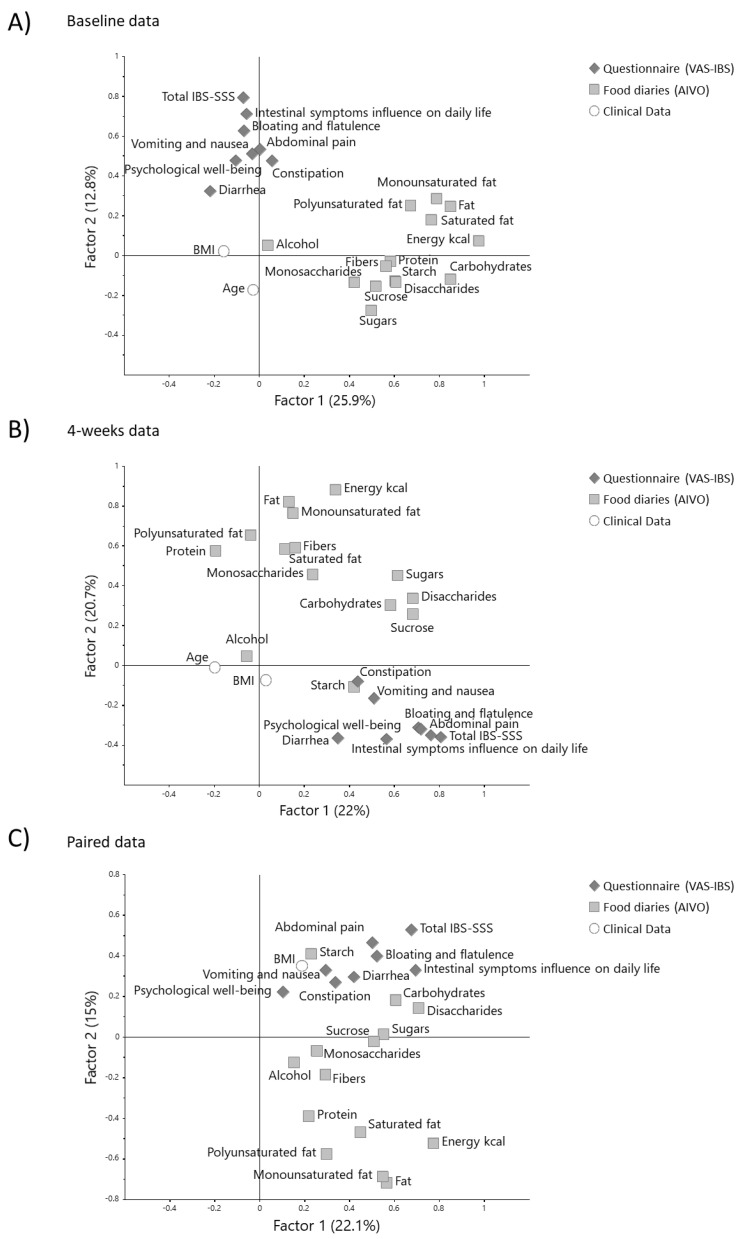
Factor analysis (2 components) for the food diaries (AIVO), questionnaires (IBS-SSS, VAS-IBS), and clinical data (age, BMI). The factor analysis was fitted for (**A**) baseline data, (**B**) 4-week data, and (**C**) paired data.

Factor analysis by the food diaries, questionnaires, and clinical data revealed that the different sources of data only correlated moderately on an individual basis (Figure 1). At the 4-week follow-up, the maximum significant correlation between the different sources of data was observed for total IBS-SSS and sucrose (*r* = 0.30) (Supplementary Figure S1). For the paired data, the maximum significant correlation between the different sources of data was observed for diarrhea and disaccharides (*r* = 0.38).

### 2.3. Metabolomic Effects of the SSRD Diet

The metabolomics data were constructed from the three different types of analysis: Gas chromatography mass spectrometry (GC-MS), liquid chromatography mass spectrometry (LC-MS) positive (+) ionization mode, and LC-MS negative (−) ionization mode. The pre-processing GC-MS data included 60 metabolic features, the LC-MS (+) included 174 metabolic features, and the LC-MS (−) included 88 metabolic features. Further, 309 of the total 322 metabolic features were uniquely annotated and detected by only one analysis.

**Figure 2:**
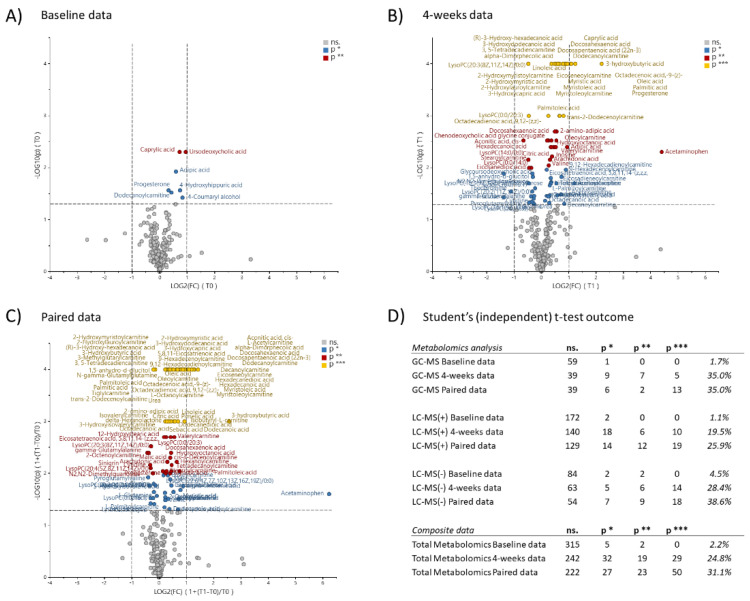
Volcano plots visualize the statistical significance (*p*) and the magnitude of change (FC) between the intervention group (*n* = 69) and the control group (*n* = 22), for the 322 metabolic features. (**A**) Baseline data, (**B**) 4-week data, (**C**) paired data, and (**D**) statistical significance testing overview for the specific types of metabolomics analysis. Statistics estimated by student’s (independent) *t*-test. Significance levels α = 0.05 (*), α = 0.01 (**), α = 0.001 (***). Positive x-axis values refer to higher average values for the intervention group.

At baseline, only seven (2.2%) out of the total 322 metabolic features indicated significant differences between the intervention group and the control group (Figure 2). According to the Human Metabolome Database (HMDB) descriptions, five out of the seven metabolic features indicated to be associated with dietary and lifestyle preferences. As an example, caprylic acid (*p* = 0.005) was linked to coconut, 4-Coumaryl alcohol (*p* = 0.038) to loquats, sweet basils, and capers, and adipic acid (*p* = 0.012) to jello products [26]. Further, progesterone (*p* = 0.027) was associated with the female menstrual cycle and pregnancy, and dodecanoylcarnitine (*p* = 0.030) can be associated with celiac disease [26]. Since only 2.2% (7 out of 322) of the metabolic features indicated significant differences at baseline, this indicates a possible spurious and random outcome. Consequently, the two groups were considered to be substantially identical at baseline, in the aspect of metabolic profiling.

All three types of metabolomics data indicated obvious associations to the diet intervention, both for the 4-week data at group level as well as for the paired individual data (Figure 2). 

#### 2.3.1. Multivariate Analysis

Since the correlations between the metabolomics data, the food diaries, questionnaires, and clinical data were weak overall, multivariate analysis (MVA) was used to assess and evaluate how well they corresponded to each other (Supplementary Table S1). In total, 72 different orthogonal partial least squares (OPLS) (1+1) models were fitted for the metabolomics data (X: *n* = 91, k = 322) against each of the food diaries, questionnaires, and clinical descriptors (Y: *n* = 91, k = 24), for all three sets of data (baseline, 4-week, and paired data). 

It was obvious that the metabolomics data were clearly associated with the clinical data. Age and BMI indicated a strong association with the metabolomics data, both at baseline and after 4 weeks. For the modeling of the paired data, the BMI was significantly linked to the differences between the intervention group and the control group, indicating increasing concentrations of 3-hydroxybutyric acid (a ketone body) with lower BMI. 

For the questionnaires data, only one descriptor indicated significant associations with the metabolomics data. Constipation was significantly modeled against the metabolomics data at baseline, indicated to be negatively associated with the levels of acylcarnitines. However, the status of constipation was relatively static during the study, which means that no changes in the metabolomics data could be linked to changes in constipation symptoms. 

For the food diaries data, some significant findings were associated with the metabolomics data. At 4 weeks, starch was significantly regressed against the metabolomics data; lowered intake of starch correlated with increment in 3-hydroxydodecanoic acid. It was also seen that polyunsaturated fat could be significantly linked to increments in lysophospholipid LysoPE(O-16:0). For the paired data, no significant models could be fitted for the metabolomics data against food diaries data (AIVO) and questionnaire data (IBS-SSS, VAS-IBS). The weak associations were due to the obvious discrepancy in specificity between the self-reporting dietary intake, i.e., macronutrient levels, and the metabolic profiling, i.e., micronutrient levels.

#### 2.3.2. Enrichment Analysis

**Figure 3:**
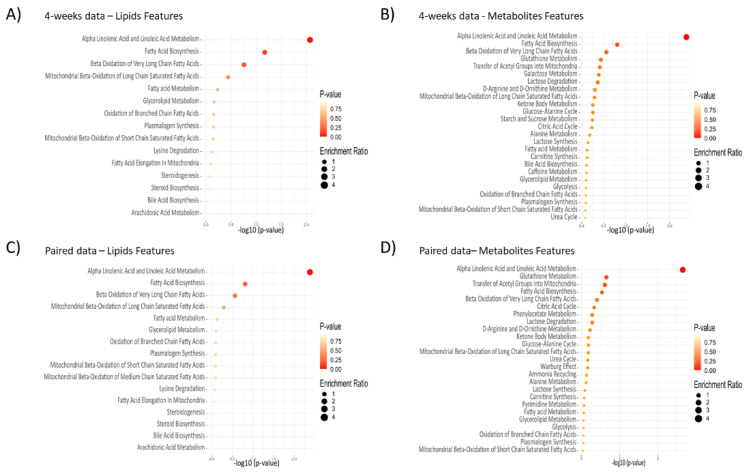
Enrichment analysis based on both metabolites and lipids feature types for the Small Molecule Pathway Database (SMPDB) normal human metabolic pathways library [27]. (**A**) 4-week data for lipid features, (**B**) 4-week data for metabolites features, (**C**) paired data for lipid features, and (**D**) paired data for metabolites features. The 4-week data included 80 significant metabolic features and the paired data included 100 significant metabolic features, out of the total 322 metabolic features (in Figure 1).

Enrichment analysis was performed based on the statistically significant metabolites according to the Student’s (independent) *t*-test, both for the 4-week follow-up data and the paired data (Figure 3). The dietary intervention effect was associated with the alpha-linolenic acid (C18:3n-3) and linoleic acid (C18:2n-6) metabolism. For the 4-week data, the major differences were associated with the main types of omega-3 fatty acids (*n*-3), such as docosahexaenoic acid (*p* < 0.001), docosapentaenoic acid (*p* < 0.001), and alpha-linolenic acid (*p* = 0.034), as well as polyunsaturated fatty acids (PUFA), such as linoleic acid (*p* < 0.001) and arachidonic acid (*p* = 0.007) (Supplementary Table S2). For the paired data, almost the same statistics were observed, with docosahexaenoic acid (*p* < 0.001), docosapentaenoic acid (*p* < 0.001), alpha-linolenic acid (*p* = 0.018), linoleic acid (*p* < 0.001), arachidonic acid (*p* = 0.002), and eicosapentaenoic acid (*p* = 0.023). The high consistency between the follow-up and paired data, both indicating an obvious change over time effect with association with linoleic acid metabolism, strengthens the connection to the dietary intervention. Further, both the fatty acid biosynthesis and the beta-oxidation of very long-chain fatty acids were associated with the dietary intervention.

For the 4-week follow-up data, 29 out of 322 metabolic features had a Student’s *t*-test *p*-value below 0.001. Out of those 29 metabolic features, 26 were characterized as either fatty acids, fatty acyls, and/or linked to fatty acid biosynthesis and metabolism. For the paired data, 50 out of 322 metabolic features had a Student’s *t*-test *p*-value below 0.001 with 42 characterized as either fatty acids, fatty acyls, and/or linked to fatty acid biosynthesis and metabolism.

## 3. Discussion

The present study aimed to link the circulating plasma metabolites (metabolomics data) to the markedly reduced bowel symptoms previously observed for IBS patients who followed the SSRD guidelines [3,20,21]. For the metabolomics data, a clear difference in trends was observed between the intervention group and the control group, which supported that the participants were indeed compliant with the general dietary advice. In addition, statistics highlighted a clear intervention effect for the daily nutrient intake (AIVO data) and the bowel symptoms (IBS-SSS and VAS-IBS questionnaire data), which supported the general success of the implementation of the intervention. Before the analysis, it was expected that the effects of the SSRD intervention were to differ in size and parity between IBS patients, as some participants already ate a more similar diet to the SSRD than others. Therefore, both independent and dependent statistical approaches were used, to take into account how much participants diverged from their own individual dietary habits at baseline. It was observed that seven of the 322 metabolic features showed significant differences at baseline but only two of those, progesterone and dodecanoylcarnitine, showed a significant effect due to the intervention (paired statistics). Since the main part of participants were women, the changes in progesterone can be linked to different sampling time points according to the menstrual cycle. The clear changes in dodecanoylcarnitine were observed by lowered levels for the SSRD group, linked to beta-oxidation and diseases like celiac disease, which can be associated with improved well-being [26].

The metabolomics data and the AIVO data indicated overall weak associations, and to investigate the relationship, a biochemical pathways enrichment approach was utilized. Hence, the metabolomics data contained micronutrient data representing the circulating blood plasma at the time point of sampling, e.g., levels of specific free fatty acid, while the AIVO data contained macronutrients representing the whole food intake on daily basis, e.g., levels of the total fat intake. The IBS patients were only guided by dietary advice and were not strictly controlled in their food consumption. Both the intervention group and the control group showed clear intra-group variability in nutrient intake, over the 4 weeks, further complicating the investigation of the link between the generalized macronutrient level and the specific micronutrient level. No obvious difference in relative variability between the baseline data and the 4-week data was observed, which infers that the nutrient data represent 91 individual diets and starting points, unique for each of the 91 IBS patients, rather than two strictly controlled diet groups. Hence, the participants bought and cooked their food according to the advice, and the dietary content differed between individuals. Noticeably, both the intervention group as well as the control group showed an overall reduction in carbohydrate intake. This reported change in nutrient intake for the control group may be due to the mandatory daily self-reporting requirements that increase participants’ awareness of their eating habits and thus change their diet unintentionally. However, the reduction in carbohydrate intake was observed to be significantly greater for the intervention group, which proves that they had a larger change in diet composition [3,21]. 

The strongest association between the metabolomics data and the AIVO data, observed after 4 weeks, was the reduced intake of starch correlating with increased levels in 3-hydroxydodecanoic acid, a medium-chain fatty acid previously linked to prolonged fasting [28]. Only weak associations were indicated between VAS-IBS data and metabolomics data, and it was mainly the constipation descriptor that was indicated to be associated with the metabolomics data at baseline. Reduced amounts of acylcarnitines were associated with a high degree of constipation for IBS patients, supported by previous research linking carnitine deficiency to constipation [29]. Although clear differences in metabolite concentrations were observed between the two groups, metabolite concentrations could not be correlated with reductions in IBS symptoms, in contrast to the correlation between metabolite concentrations and GI symptoms as have been found in previous cross-sectional studies [8,9,10]. In addition, variation in the intervention group was expected as some patients with IBS experience worse symptoms of fat, fiber, and dairy products [30], food items that were more ingested during the SSRD regime [3,22]. Unabsorbed carbohydrates might lead to increased small intestinal gas and water volume, causing bowel symptoms, further exacerbated by visceral hypersensitivity in IBS patients [31]. Similar to our present SSRD trial, a previous dietary intervention with low fermentable oligosaccharides, disaccharides, and monosaccharides and polyols (FODMAP) showed changes in urine metabolites after the intervention, without being able to correlate the changes to specific food items or symptoms [32].

For the metabolomics data and the biochemical pathway enrichment approach, the most obvious circulating plasma metabolites linked the present SSRD intervention effect to linoleic acid metabolism, fatty acid biosynthesis, and beta-oxidation. By the statistical tests performed for each of the 322 metabolic features, the top metabolic findings linked the SSRD intervention to significantly increased levels of the two known essential free fatty acids for humans, i.e., alpha-linoleic acid and linoleic acid. Essential metabolites cannot be synthesized fast enough, and therefore the uptake mainly occurs through diet. Fatty acids are expected to be found in higher concentrations in animal products, vegetables, and fruits, compared to many of the starch and sucrose-rich food sources like cereals, potatoes, and sugar beets. By following the SSRD recommendations with a reduction in the carbohydrate intake in favor of increased intake of specific fruits and vegetables [22], it was expected to increase the relative levels of the free fatty acids in the circulating blood plasma. Due to the increments in alpha-linoleic acid and linoleic acid, commonly associated with fish consumption, it was indicated that the intervention group went for what they consider to be a healthier food choice and did not just change the part specified by the SSRD dietary advice. The more detailed levels of the carbohydrate data, i.e., the reductions in starch, disaccharides, and sucrose intake for the intervention group, further strengthen the associations between reduced intake of starch- and sucrose-rich food sources with increased intake of the recommended SSRD food sources [22].

Some studies have linked IBS with low-grade inflammation and the infiltration of proinflammatory cytokines and tumor necrosis factor alpha (TNF-alpha) in the colonic mucosa, which may lead to exacerbated IBS symptoms [33]. Of interest is that PUFA has been linked with IBS on an epidemiological level in the EPIC-Norfolk cohort, where they highlighted a protective effect from PUFA intake, specifically oleic acid (a precursor of linoleic acid) [34]. In the present study, we showed an increase in linoleic acid and alpha-linoleic acid after the diet intervention. Both linoleic and alpha-linoleic acid are considered essential fatty acids since humans lack the enzymes needed for their synthesis. Additionally, linoleic and alpha-linoleic acid are precursors of the biologically important arachidonic acid, eicosapentaenoic acid, and docosahexaenoic acid. To this end, mechanistic models show reduced inflammation with linoleic-supplementation via downregulation of tumor necrosis factor alpha (TNF-alpha) and nuclear factor kappaB (NF-kB) and increased transforming factor beta (TGF-B) and peroxisome proliferator-activated receptor-gamma (PPAR-y) [35]. Thus, the increase in linoleic metabolites from a starched-reduced dietary intervention may contribute to a reduced inflammatory state locally in the GI tract. However, circulating cytokine levels were not affected by the SSRD [21], and local inflammation as a general genesis to IBS has never been possible to confirm [1]. 

The clinical relevance of the present study is that we can assure a good adherence to the dietary advice given, and the observed effect in improvement of GI symptoms and weight may therefore be ascribed to the dietary changes.

## 4. Materials and Methods

The study was approved by the Ethical Review Board of Lund University (2017/171, date of approval: 27 April 2017, and 2017/810, date of approval: 16 October 2017) and performed in accordance with the declaration of Helsinki at Skåne University Hospital, Malmö, Sweden. All subjects gave their written, informed consent before inclusion in the study. The study was registered at the ClinicalTrials.gov database (NCT03306381).

### 4.1. Study Design

The study design is a randomized, open clinical trial with a dietary intervention for 4 weeks (Supplementary Figure S2). Patients with an IBS diagnosis completed a study questionnaire addressing socioeconomic factors, lifestyle habits, health declaration, the Rome IV questionnaire, the IBS-SSS, the VAS-IBS, and a food diary during day 6–10 of the run-in period. Subjects who suffered from abdominal pain at least once weekly along with altered bowel habits and had a score of ≥175 of the IBS-SSS, without any exclusion criteria, were included in the study. Patients randomized to serve as controls kept their ordinary food habits during the observational time, whereas the patients randomized to the intervention group received information about SSRD. At the end of the intervention, participants once again completed the food diaries (day 24–28), as well as the Rome IV questionnaire, IBS-SSS, and VAS-IBS (day 28) (Supplementary Figure S3). Blood samples were drawn before and after the study start and plasma was separated. Plasma was kept frozen at −80 °C until further analyzed for metabolomics.

### 4.2. Patients

Inclusion criteria for the study were a diagnosis of IBS, age 18–70 years, and Northern European heritage. Exclusion criteria were insufficient symptoms, <175 scores on IBS-SSS, presence of any organic GI disease, severe organic, and psychiatric diseases, or already on a diet (Supplementary Figure S3). 

A patient registry was provided by Region Skåne, which registered all subjects who had received an IBS diagnosis code of K580 or K589 (according to the International Statistical Classification of Diseases and Related Health Problems–ICD-10) in primary health care centers (PCC) during 2015–2017. Another registration of all patients who had received the diagnosis IBS, K580, or K589, from the Department of Gastroenterology and Hepatology during 2016–2017 was provided. 

In total, 1039 unique IBS patients from PCC were identified. Invitation letters were randomly sent to 528 patients after exclusion of all patients with names suggesting an ethnicity outside Scandinavia/Northern Europe, patients living outside the closest neighborhood of the cities Lund and Malmö, or patients whose telephone numbers could not be found. From the tertiary care center, 640 unique patients were identified. Invitation letters were sent to 151 patients according to the same criteria as stated above. The patients were contacted by phone a couple of weeks later. After further information, 145 patients were willing to participate (112 patients (77%) from PCC; 34 males (23%)) in the study and visit an appointment at the Internal Medicine Research Group, Skåne University Hospital, Malmö. Reasons not to participate were published previously [3,20,21]. After acceptance to participate, 40 patients (11 men (28%)) were excluded because they did not show up or were not willing to participate at a later time point (*n* = 18), had mild symptoms (*n* = 14), wrong diagnoses (*n* = 5) or were already on a diet (*n* = 3). Thus, 105 patients (23 males (22%)) were finally included in the study (77 patients (73%) from the PCC) from the 679 invitation letters sent (15% inclusion rate). Of these, 91 participants completed the study with available, measurable plasma samples from before and after the study start (Supplementary Figure S2). Seventeen IBS patients (18.7%) suffered from functional gastrointestinal disorders (FGID) with abdominal pain at least once weekly and IBS-SSS > 175 but did not fulfill the IBS criteria [1]. Of the remaining patients, 29 patients (31.9%) had mixed IBS (IBS-M), 23 patients (25.3%) had diarrhea-predominant IBS (IBS-D), and 19 patients (20.9%) had constipation-predominated IBS (IBS-C), and three patients had unspecified IBS.

### 4.3. Dietary Advice

The patients were instructed to follow a diet with starch and sucrose restriction, according to the advice given to patients with CSID [22]. Briefly, all forms of sucrose-containing food, e.g., candies, cakes, jam, and juice, should be excluded and replaced by nuts in the case of sweet cravings. The starch content was reduced with less intake of cereals, but more intake of meat, fish, egg, and dairy products. Fruits and vegetables with low starch content were recommended (Supplementary Table S3). The content of gluten and lactose was unrestricted. Fiber-rich bread, raw rice, and fiber-rich pasta were preferred instead of white bread and ordinary rice and pasta, to delay the nutrient transport through the GI tract. Participants were still recommended to restrict their intake of fiber-rich cereals to a maximum of one serving per day. Adding fat (e.g., avocado, olive oil) and/or protein to starch-rich foods was also recommended to further delay GI transport and enhance starch tolerance through long exposure to digestive intestinal enzyme activity. The patients were encouraged to eat slowly and chew their food properly, to increase the secretion of amylase, which can contribute to the degradation of starch. Participants in the control group received no dietary advice and were urged not to make any changes to their ordinary diet. 

All participants were encouraged to continue with their ordinary energy intake, degree of physical activity, and medications, without making any changes. If they used any form of probiotics or were on a diet not excluding them from enrollment, they had to continue with this during the study, without introducing any new drugs or other dietary changes. The participants could reach the study staff by telephone or email, whenever they wanted during the study.

### 4.4. Questionnaires

#### 4.4.1. Study Questionnaire

A study questionnaire about sociodemographic factors, family history, lifestyle habits, medical health, and pharmacological treatment was completed before the study start.

#### 4.4.2. Food Diaries

Participants were instructed to register all consumed foods in a free writing structure, with the requirement to provide information on time/type of food intake and GI symptoms about food intake for 4 days, before and at the end of the dietary intervention. The patients reported the amount and/or volume of each food item, including the percentage of fat in dairy products, fiber in bread products, and cacao in chocolate, as well as information on the type of soda (sugar-free or regular) consumed. The manufacturer of the product was given when applicable, e.g., for brands of bread, butter, and muesli. The product name and ingredient list for pre-maid dishes were reported. For each patient, nutrient intake was calculated from day 2 of the 4-day registrations. Daily nutrient intake calculations, in total amounts of grams, were performed by a nutritionist, using the AIVO Diet computer program from the National Food Agency, Sweden [23].

#### 4.4.3. Rome IV Questionnaire

The Rome IV questionnaire is developed to diagnose functional gastrointestinal disorders (FGID) [36]. Questions No 40–48 in the Swedish version of the questionnaire were used, after having received a license from The Rome Foundation, Inc. Raleigh, NC, USA.

#### 4.4.4. Irritable Bowel Syndrome-Symptom Severity Score

IBS-SSS uses visual analog scales (VAS) to estimate abdominal pain, abdominal distension, satisfaction with bowel habits, and the impact of bowel habits on daily life. Information was also collected on the number of days with abdominal pain in the last 10 days. The VAS ranges from 0–100 where a score of 0 mm implies “no symptoms”, and a score of 100 mm implies “severe symptoms”. Combining the 5 question areas, the maximum achievable score is 500. Scores ranging from 75–174 indicate mild disease, 175–299 indicate moderate disease and ≥300 indicate severe disease [24].

#### 4.4.5. The Visual Analog Scale for Irritable Bowel Syndrome Questionnaire

The VAS-IBS is a validated questionnaire covering abdominal pain, diarrhea, constipation, bloating and flatulence, vomiting and nausea, psychological well-being, and intestinal symptoms’ influence on daily life. Items are measured on a scale of 0–100 mm, ranging from absent (0 mm) to very severe (100 mm) symptoms. The values are inverted from the original format. The questionnaire is validated to measure changes over time [25].

### 4.5. Metabolomics

Plasma collected before and after the intervention was examined at the Swedish Metabolomic Center, Umeå. The UHPLC-MS analysis was performed with an Infinity 1290 Agilent (Agilent Technologies, Santa Clara, CA, USA) ultra-high performance liquid chromatograph coupled with tandem mass spectrometry (UHPLC-MSMS) as previously described in detail [37]. The GC-MS analysis was performed with Agilent 6890 GC equipment and a fused silica capillary column (10 m × 0.18 mm I.D.) with a chemically bonded 0.18 µm DB5-MS stationary phase (J&W Scientific, Folsom, CA, USA) as previously described [38]. 

Targeted feature extraction of the acquired UHPLC-MS data was performed using the MassHunter Profinder software, version B.08.00 (Agilent Technologies, Santa Clara, CA, USA). In-house libraries with exact masses and experimental retention times were used for identification. The libraries contained metabolites from the chemical classes acylcarnitines, amino acids, carbohydrates, fatty acids, bile acids, nucleotides, small peptides, and lysophospholipids. The positive ionization mode for metabolite identification of species was +H, +Na, +K, and +NH4, and the negative ionization mode was –H and –HCOO. The mass tolerance was 10 ppm, and the retention time tolerance was 0.1 min. Only one charge was allowed for each metabolite. The extracted peaks were aligned and matched between samples, and each compound was manually checked for mass and retention time agreement with the library. A two-step filtering approach was used for peak quality control. First, peaks with bad characteristics (e.g., overloaded, sample noise, non-Gaussian) were excluded from the analysis. Second, only peaks present in at least 75% of at least one study group were included. 

Raw GC-MS data files were exported in NetCDF format to MATLAB Release 2016a (Mathworks, Natick, MA, USA) where baseline correction, chromatogram alignment, and peak deconvolution were performed. Metabolite annotation was performed based on the retention index (RI) values and MS spectra from the in-house mass spectra library established. The total number of annotated metabolites by UHPLC-MS and GC-MS were 262 and 60, respectively. There was a small overlap in annotations between the three analyses, with 309 unique annotations of the total 322 metabolic features. All metabolomics data were relative concentrations.

### 4.6. Statistical Analysis

For each data source, three sets of data were generated: Baseline data (T0), 4-week data (T1), and paired data (1+(T1−T0)/T0). Statistical tests were performed for comparisons between the intervention group (*n* = 69) and the control group (*n* = 22). SPSS (IBM Corp. Released 2020. IBM SPSS Statistics for Windows, Version 27.0. Armonk, NY, USA: IBM Corp) was used for Student’s (independent-samples) *t*-test, Levene’s test, Factor Analysis, and Bivariate Correlations (Pearson’s r). GraphPad Prism (version 9.1.0 for Windows, GraphPad Software, San Diego, CA, USA) was used for Contingency tables analysis by either Chi-square or Fisher’s exact test. For the descriptive statistics, the population data were described by mean ± standard deviation, minimum to maximum value range, and counting. Multivariate analysis (MVA) was used for modeling the relationship between the intervention group and the control group. SIMCA-P+ (ver 16.0.0.7738, 64-bit, Sartorius Stedim Data Analytics AB, Umeå, Sweden) was used for OPLS modeling of the clinical, food diaries, and questionnaire data against the metabolomics data. For the statistical tests, the significance levels α = 0.05 (*), α = 0.01 (**), α = 0.001 (***) were used. Metaboanalyst was used for the enrichment analysis of the annotated metabolic features [39]. The Small Molecule Pathway Database (SMPDB) was used for the enrichment pathway modeling [27]. The Human Metabolome Database (HMDB) was used for information about biological and chemical properties [26].

## 5. Conclusions

In conclusion, the present study has proved that a dietary change with reduced starch and sucrose intake leads to marked changes in circulating metabolite concentrations at the group level. It was obvious that there was a clear discrepancy between the food diaries, collecting data on the macronutrient level, and the metabolomics analysis, collecting data on the micronutrient level. The marked reduction of GI symptoms observed could not be explained by changes in circulating metabolomics. Future studies with comparisons of SSRD with other diets may identify the mechanisms behind the improvement of IBS symptoms. Nevertheless, the diet and nutrient content seems to be of great importance for the symptom development in IBS.

## Figures and Tables

**Table 1:** Basal characteristics, daily intake, symptoms (irritable bowel syndrome- symptom severity scale, IBS-SSS, and visual analog scale for irritable bowel syndrome, VAS-IBS), concomitant diseases, and drugs in the study population. Nutrient levels were calculated from day 2 of the 4-day food diary registration before and at the end of the 4-week dietary intervention with the AIVO Diet computer program [23]. The statistical significance (Sig., *p*) was estimated by student’s (independent) *t*-test, Chi-square test, or Fisher´s exact test. Significance levels α = 0.05 (*), α = 0.01 (**), α = 0.001 (***). The paired data were calculated as the relative individual change, for the baseline data and the 4-week data. Data were expressed by either the mean ± the standard deviation or the count (percentage).

	Control Group (*n* = 22)			Intervention Group (*n* = 69)			Sig. (*p*)	
Age (year)	41.65 ± 15.03			48.49 ± 12.51			0.058	ns.
Disease duration (year)	18.81 ± 13.83			21.33 ± 13.84			0.523	ns.
Women (*n*, %)	19 (86.4)			52 (75.4)			0.381	ns.
Active Smokers (*n*, (%))	4 (18.2)			5 (7.2)			0.212	ns.
Diseases (*n*, %)								
*Allergy*	5 (22.7)			9 (13.0)			0.314	ns.
*Hypothyroid disease*	4 (18.2)			6 (8.7)			0.247	ns.
*Asthma bronchialis*	3 (13.6)			8 (11.6)			0.723	ns.
*Hypertension*	3 (13.6)			7 (10.1)			0.699	ns.
*Depression*	2 (9.1)			5 (7.2)			0.674	ns.
*Migraine*	0 (0)			4 (5.8)			0.569	ns.
Drug treatment (*n*, (%))								
*Antidepressants*	4 (18.2)			11 (15.9)			0.752	ns.
*Levaxine*	4 (18.2)			7 (10.1)			0.451	ns.
*Vitamin D*	4 (18.2)			7 (10.1)			0.451	ns.
*Asthma inhalators*	3 (13.6)			3 (4.3)			0.150	ns.
*Laxatives*	2 (9.1)			9 (13.0)			1	ns.
*Proton pump inhibitor*	2 (9.1)			9 (13.0)			1	ns.
*Hormonal treatment*	2 (9.1)			6 (8.7)			1	ns.
*Folic acid*	2 (9.1)			3 (4.3)			0.591	ns.
*Cobalamin*	2 (9.1)			3 (4.3)			0.591	ns.
*Statins*	1 (4.5)			5 (7.2)			1	ns.
Physical activity (*n*, (%))							0.730	ns.
*No activity*	1 (4.5)			9 (13.0)				
*<30* min	5 (22.7)			15 (21.7)				
*30–60* min	4 (18.2)			11 (15.9)				
*60–90* min	3 (13.6)			6 (8.7)				
*90–120* min	2 (9.1)			12 (17.4)				
*>120* min	7 (31.8)			16 (23.2)				
	**Baseline data**	**4-week data**	**Paired data**	**Baseline data**	**4-week data**	**Paired data**		
BMI (kg/m^2^)			1.02 ± 0.06			0.972 ± 0.023	0.002	(**)
		25.39 ± 3.75			25.32 ± 4.54		0.945	ns.
	25.1 ± 3.87			25.9 ± 4.597			0.471	ns.
**Food diaries (AIVO)**								
Carbohydrates (g)			0.921 ± 0.49			0.599 ± 0.388	0.002	(**)
	194.9 ± 78.31	169.3 ± 79.7		193.9 ± 64.52	104 ± 55.03			
Starch (g)			1.178 ± 0.973			0.447 ± 0.542	0.002	(**)
	79.34 ± 41.35	73.84 ± 43.25		82.62 ± 42.11	30.87 ± 31.67			
Disaccharides (g)			0.965 ± 0.708			0.544 ± 0.466	0.014	(*)
	45.53 ± 36.5	35.83 ± 23.93		41.76 ± 23.56	18.98 ± 15.4			
Sucrose (g)			0.966 ± 0.902			0.524 ± 0.851	0.040	(*)
	34.42 ± 34.41	22.66 ± 17.24		29.68 ± 21.28	10.15 ± 12.79			
Fat (g)			1.182 ± 0.537			1.384 ± 0.843	0.190	ns.
	65.35 ± 27.67	70.74 ± 28.2		73.12 ± 36.42	85.16 ± 45.67			
Monounsaturated fat (g)			1.253 ± 0.783			1.595 ± 1.367	0.278	ns.
	24.45 ± 11.22	26.49 ± 12.37		27.76 ± 14.62	33.90 ± 22.25			
Saturated fat (g)			1.148 ± 0.643			1.326 ± 0.928	0.415	ns.
	27.35 ± 13.63	28.77 ± 15.96		27.63 ± 16.65	29.07 ± 19.55			
Monosaccharides (g)			1.015 ± 0.885			1.295 ± 1.526	0.415	ns.
	24.66 ± 12.83	20.19 ± 14.53		24.81 ± 14.23	22.02 ± 16.62			
Fibre (g)			0.976 ± 0.361			1.06 ± 0.59	0.427	ns.
	17.46 ± 6.99	16.92 ± 9.27		20.45 ± 10.34	18.36 ± 8.09			
Energy (kcal)			1.001 ± 0.341			0.941 ± 0.372	0.502	ns.
	1696 ± 528.4	1641 ± 538.5		1797 ± 594.9	1569 ± 549.6			
Protein (g)			1.198 ± 0.553			1.253 ± 0.579	0.692	ns.
	64.91 ± 27.52	70.61 ± 24.72		72.89 ± 24.32	82.93 ± 30.02			
Polyunsaturated fat (g)			1.442 ± 0.748			1.535 ± 1.025	0.701	ns.
	8.150 ± 3.580	10.00 ± 3.761		10.74 ± 5.303	13.64 ± 8.051			
Sugars (g)			0.893 ± 0.578			0.838 ± 0.697	0.737	ns.
	73.62 ± 44.59	58.78 ± 38.52		71.53 ± 37.54	47.45 ± 32.44			
Alcohol (g)			0.862 ± 0.317			0.846 ± 0.395	0.867	ns.
	5.386 ± 13.17	2.914 ± 5.629		4.235 ± 8.82	3.648 ± 12.92			
**Questionnaire (IBS-SSS, VAS-IBS)**								
Total IBS-SSS			0.965 ± 0.223			0.561 ± 0.352	<0.001	(***)
		288.6 ± 78.14			168.5 ± 104.4		<0.001	(***)
	302.1 ± 62.59			301.1 ± 73.5			0.953	ns.
Abdominal pain			1.044 ± 0.377			0.58 ± 0.485	<0.001	(***)
	50.73 ± 22.88	49.55 ± 20.19		49.28 ± 20.44	27.29 ± 24.07			
Bloating and flatulence			0.843 ± 0.246			0.501 ± 0.436	<0.001	(***)
	76.86 ± 21.61	65 ± 22.64		68.71 ± 25.12	33.74 ± 27.91			
Intestinal symptoms influence on daily life			1.01 ± 0.274			0.664 ± 0.58	<0.001	(***)
	65.05 ± 16.21	64 ± 19.1		65.99 ± 22.53	39.5 ± 27.36			
Diarrhea			1.086 ± 1.188			0.491 ± 0.523	0.032	(*)
	35.86 ± 32.22	27.55 ± 28.19		47 ± 31.92	21.61 ± 25.21			
Constipation			0.775 ± 0.661			0.565 ± 0.421	0.082	ns.
	49.05 ± 28.09	32.77 ± 32.14		41.62 ± 35.15	22.28 ± 24.05			
Vomiting and nausea			1.062 ± 1.866			0.748 ± 0.914	0.29	ns.
	27.91 ± 23.9	23.91 ± 27.5		23.23 ± 26.28	13.87 ± 21.06			
Psychological well-being			1.152 ± 0.732			1.068 ± 1.967	0.845	ns.
	47.32 ± 26.51	45.27 ± 21.6		47.13 ± 25.5	35.97 ± 24.63			

## Data Availability

The data presented in this study are available on request from the corresponding author. The data are not publicly available due to data protection regulation law.

## Supplementary Materials

The following are available online at https://www.mdpi.com/article/10.3390/metabo11070440/s1, Figure S1: Correlation matrix for food diaries, questionnaires, and clinical data. Table S1: Metabolomics modeling against food diaries, questionnaire, and clinical data. Table S2: Statistically significant metabolic features linking the Metaboanalyst pathway enrichment and Small Molecule Pathway Database output with the Human Metabolome Database descriptions. Figure S2: CONSORT 2010 flow diagram. Figure S3: Flow chart over inclusion and exclusion criteria. Table S3: Starch- and sucrose-reduced diet (SSRD) recommendations.
