# Supplementary material for: Metabolic Profiling of Plasma in Patients with Irritable Bowel Syndrome after a 4-Week Starch- and Sucrose-Reduced Diet
Source: Metabolites. 2021 Jul 4;11(7):440. doi: 10.3390/metabo11070440 (PMC8306703; doi:10.3390/metabo11070440)
Supplement: Supplementary file 1 [file metabolites-11-00440-s001.zip › metabolites-1283752-supplementary(1).pdf]

metabolites

Supplementary Materials

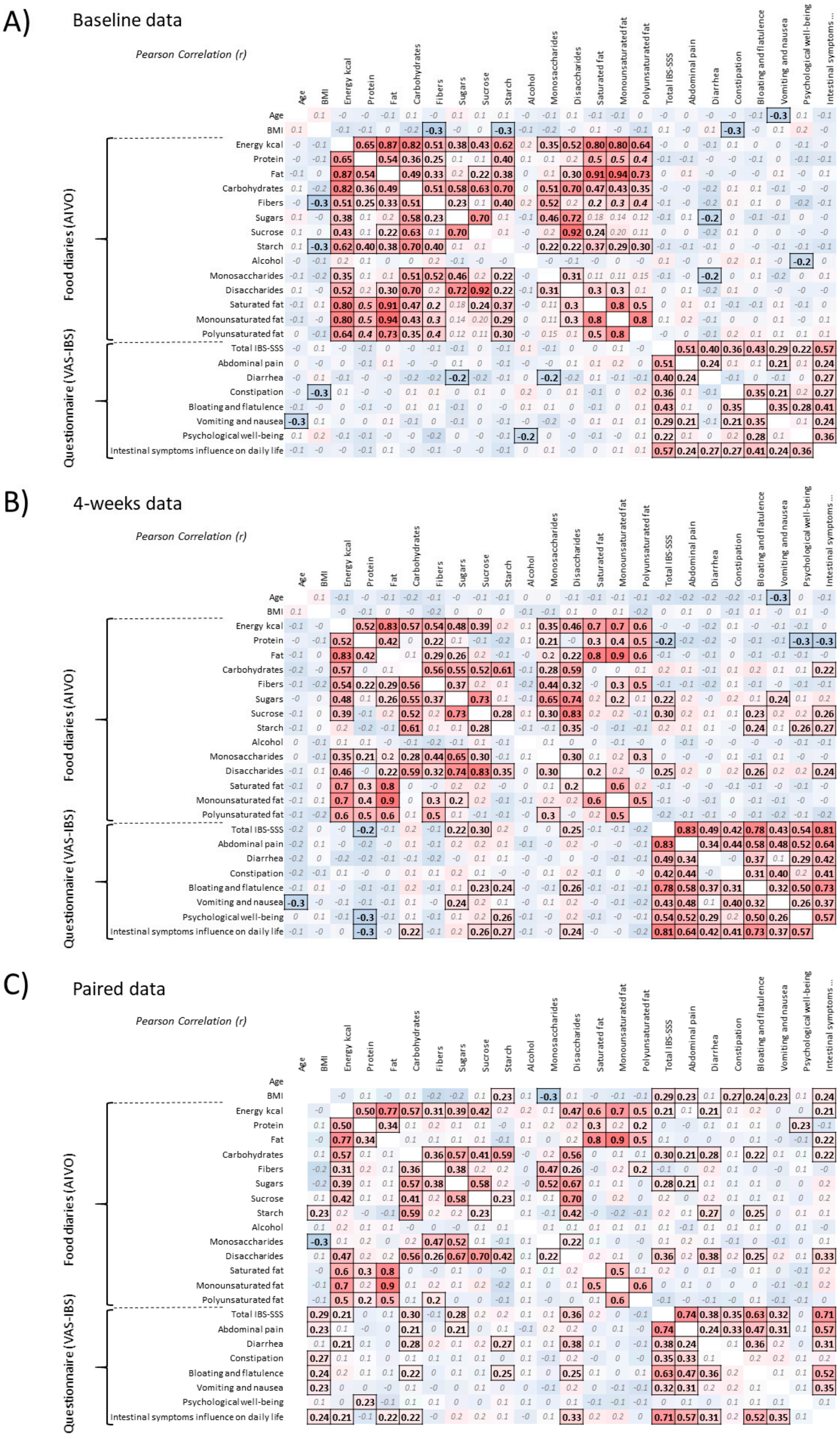

Supplementary Figure 1. Correlation matrix (Pearson, r) for food diaries (AIVO), questionnaires (IBS-SSS, VAS-IBS), and clinical data (age, BMI). The correlation matrices were calculated for A)

baseline data, B) 4-week data, and C) paired data. Significance level α = .05. Significant correlations are denoted by bold text and borders. Blue-red colors according to the Pearson correlation coefficient (r), blue color denotes negative correlations and red color denotes positive correlations.

Supplementary Table 1. Metabolomics modeling against food diaries, questionnaire, and clinical data. Orthogonal partial least squares (OPLS) (1+1) models regressing the metabolomics data (n=91, k=322) against each one of the food diaries (AIVO), questionnaires (IBS-SSS, VAS-IBS), and clinical data (age, BMI) descriptors (n=91, k=24). The paired data was calculated as the relative individual change between the baseline data and the 4-week data. The CV-ANOVA p-values refer to OPLS model statistics. Significance levels α = .05 (*), α = .01 (**), α = .001 (***) was used. The OPLS p1-loadings were reported for the metabolic feature with maximum corresponding predictive VIP value. In addition, the maximum absolute correlation (Pearson, r) was reported.

OPLS (1+1) Model (X=322 metabolic features, N=91)	R2X(cum)	R2Y(cum)	Q2(cum)	p (CV-ANOVA)		Max. VIP (pred.) Metabolite	p-loadings	Max. Correlation Metabolite	Pearson (r)
**Baseline data - Age**	**0.233**	**0.685**	**0.454**	**1.01E-10**	***(***)***	**Inositol, Myo-**	**0.123**	**L-Palmitoylcarnitine**	**0.564**
**Baseline data - BMI**	**0.219**	**0.609**	**0.209**	**2.71E-05**	***(***)***	**LysoPC(19:0/0:0)**	**-0.175**	**Uric acid**	**0.496**
Baseline data - Energy kcal	0.213	0.547	-0.11	1	*ns.*	Cholic acid	0.181	Deoxycholic acid	0.402
Baseline data - Protein	0.22	0.488	-0.189	1	*ns.*	Valine	0.175	Tiglylcarnitine	0.363
Baseline data - Fat	0.207	0.522	-0.203	1	*ns.*	Deoxycholic acid	0.227	Deoxycholic acid	0.479
Baseline data - Carbohydrates	0.215	0.523	-0.133	1	*ns.*	LysoPC(12:0/0:0)	0.166	Dodecanoic acid	0.322
Baseline data - Fibers	0.192	0.539	0.0385	0.492	*ns.*	2,4-Dihydroxybenzoic acid	0.228	Dodecanedioic acid	0.399
Baseline data - Sugars	0.226	0.415	-0.104	1	*ns.*	Palmitoleic acid	0.204	Palmitoleic acid	0.314
Baseline data - Sucrose	0.219	0.413	-0.358	1	*ns.*	Myristic acid	0.204	Phenylalanylglutamic acid	0.290
Baseline data - Starch	0.227	0.447	-0.239	1	*ns.*	Octadecenoic acid,-9-(z)-	-0.176	LysoPC(0:0/15:0)	0.328
Baseline data - Alcohol	0.207	0.466	0.0356	0.406	*ns.*	Inositol, Scyllo-	0.192	3-indolepropionic acid	0.472
Baseline data - Monosaccharides	0.218	0.465	-0.0571	1	*ns.*	LysoPC(0:0/19:0)	0.165	Tetradecanedioic acid	0.303
Baseline data - Disaccharides	0.215	0.463	-0.356	1	*ns.*	Myristic acid	0.218	Deoxycholic acid	0.273
Baseline data - Saturated fat	0.212	0.576	-0.0981	1	*ns.*	Cholic acid	0.250	Deoxycholic acid	0.577
Baseline data - Monounsaturated fat	0.103	0.536	-0.25	1	*ns.*	Deoxycholic acid	0.220	Deoxycholic acid	0.338
Baseline data - Polyunsaturated fat	0.217	0.425	-0.404	1	*ns.*	Xanthine	0.182	Xanthine	0.301
Baseline data - Total IBS-SSS	0.134	0.65	-0.0797	1	*ns.*	Citric acid	-0.236	Citric acid	-0.405
Baseline data - Abdominal pain	0.202	0.519	-0.203	1	*ns.*	Phenylalanine	-0.176	Homocitrulline	-0.339
Baseline data - Diarrhea	0.106	0.571	-0.0444	1	*ns.*	Citric acid	-0.223	Citric acid	-0.377
**Baseline data - Constipation**	**0.219**	**0.478**	**0.114**	**0.0320**	***(*)***	**2-Hydroxymyristoylcarnitine**	**-0.176**	**Propionylcarnitine**	**-0.380**
Baseline data - Bloating and flatulence	0.227	0.362	-0.227	1	*ns.*	LysoPC(20:2(11Z,14Z)/0:0)	0.159	Docosatetraenoylcarnitine	-0.292
Baseline data - Vomiting and nausea	0.214	0.489	-0.351	1	*ns.*	Adenosine	0.187	Tartaric acid	0.323

Baseline data - Psychological well-being	0.224	0.437	-0.155	1	*ns.*	Cytidine	0.159	Cytidine	0.339
Baseline data - Intestinal symptoms influence on daily life	0.191	0.441	-0.258	1	*ns.*	LysoPC(18:1(9Z)/0:0)	0.200	Tyrosine	-0.293

OPLS (1+1) Model (X=322 metabolic features, N=91)	R2X(cum)	R2Y(cum)	Q2(cum)	p (CV-ANOVA)		Max. VIP (pred.) Metabolite	p-loadings	Max. Correlation Metabolite	Pearson (r)
**4-weeks data - Age**	**0.228**	**0.665**	**0.412**	**2.19E-09**	***(***)***	**gamma-Glutamyltyrosine**	**0.133**	**Ornithine**	**0.571**
**4-weeks data - BMI**	**0.167**	**0.705**	**0.323**	**7.58E-07**	***(***)***	**L-Glutamic acid**	**0.199**	**Glucose**	**0.495**
4-weeks data - Energy kcal	0.229	0.365	-0.251	1	*ns.*	Oleoylcarnitine	-0.217	Octadecenoic acid,-9-(z)-	-0.275
4-weeks data - Protein	0.213	0.443	-0.015	1	*ns.*	Valine	0.225	Valine	0.334
4-weeks data - Fat	0.21	0.506	-0.217	1	*ns.*	12-Hydroxystearic acid	0.189	12-Hydroxystearic acid	0.330
4-weeks data - Carbohydrates	0.15	0.447	-0.0405	1	*ns.*	LysoPC(0:0/14:0)	0.147	3-Hydroxybutyric acid	-0.380
4-weeks data - Fibers	0.175	0.463	-0.0374	1	*ns.*	2,4-Dihydroxybenzoic acid	0.199	2,4-Dihydroxybenzoic acid	0.359
4-weeks data - Sugars	0.212	0.393	-0.374	1	*ns.*	Valine	-0.171	L-threonic acid	0.267
4-weeks data - Sucrose	0.139	0.424	-0.236	1	*ns.*	Valine	-0.214	L-threonic acid	0.318
**4-weeks data - Starch**	**0.166**	**0.575**	**0.149**	**0.00726**	***(**)***	**3-Hydroxydodecanoic acid**	**-0.144**	**4-Guanidinobutanoic acid**	**0.403**
4-weeks data - Alcohol	0.206	0.457	-0.258	1	*ns.*	Palmitoleic acid	-0.213	Malic acid	0.314
4-weeks data - Monosaccharides	0.225	0.417	-0.414	1	*ns.*	Xanthine	0.224	Xanthine	0.422
4-weeks data - Disaccharides	0.142	0.412	-0.253	1	*ns.*	Cervonylcarnitine	-0.161	2-Hydroxymyristoylcarnitine	-0.276
4-weeks data - Saturated fat	0.215	0.572	-0.312	1	*ns.*	2-amino-adipic acid	0.179	Isoleucyl-Leucine	0.394
4-weeks data - Monounsaturated fat	0.212	0.484	-0.15	1	*ns.*	LysoPE(O-16:0/0:0)	0.201	LysoPE(P-16:0/0:0)	0.360
**4-weeks data - Polyunsaturated fat**	**0.216**	**0.504**	**0.106**	**0.0468462**	***(*)***	**LysoPE(O-16:0/0:0)**	**0.205**	**LysoPE(O-16:0/0:0)**	**0.378**
4-weeks data - Total IBS-SSS	0.21	0.466	-0.0215	1	*ns.*	LysoPC(20:0/0:0)	0.181	Propionylcarnitine	-0.302
4-weeks data - Abdominal pain	0.216	0.453	-0.0831	1	*ns.*	Propionylcarnitine	-0.177	Adipic acid	-0.309
4-weeks data - Diarrhea	0.21	0.466	-0.192	1	*ns.*	Myristoleic acid	0.201	Abietic acid	0.273
4-weeks data - Constipation	0.191	0.467	0.0945	0.071	*ns.*	2-Hydroxymyristoylcarnitine	-0.179	2-Hydroxylauroylcarnitine	-0.412
4-weeks data - Bloating and flatulence	0.141	0.525	0.0765	0.140	*ns.*	3-Hydroxyisovaleric acid	-0.172	Eicosanedioic acid	0.397
4-weeks data - Vomiting and nausea	0.216	0.516	-0.0514	1	*ns.*	Eicosatetraenoic acid, 5,8,11,14-(z,z,z,z,)-	-0.192	Sulfolithocholylglycine	-0.328
4-weeks data - Psychological well-being	0.208	0.473	-0.161	1	*ns.*	2-Hydroxylauroylcarnitine	-0.231	2-Hydroxylauroylcarnitine	-0.273

4-weeks data - Intestinal symptoms influence on daily life	0.143	0.525	0.0304	0.478	*ns.*	LysoPC(20:0/0:0)	0.173	2-Hydroxylauroylcarnitine	-0.358

OPLS (1+1) Model (X=322 metabolic features, N=91)	R2X(cum)	R2Y(cum)	Q2(cum)	p (CV-ANOVA)		Max. VIP (pred.) Metabolite	p-loadings	Max. Correlation Metabolite	Pearson (r)
**Paired data - BMI**	**0.195**	**0.623**	**0.15**	**0.00749073**	***(**)***	**3-Hydroxybutyric acid**	**-0.147**	**Cholic acid**	**0.652**
Paired data - Energy kcal	0.172	0.571	-0.488	1	*ns.*	Progesterone	-0.182	Glyceric acid	0.352
Paired data - Protein	0.108	0.588	-0.154	1	*ns.*	Sebacic acid	0.258	Glycoursodeoxycholic acid	0.325
Paired data - Fat	0.177	0.514	-0.376	1	*ns.*	LysoPC(20:5(5Z,8Z,11Z,14Z,17Z)/0:0)	0.184	LysoPC(20:0/0:0)	0.305
Paired data - Carbohydrates	0.186	0.541	-0.0833	1	*ns.*	2-amino-adipic acid	-0.158	Niacinamide	0.401
Paired data - Fibers	0.149	0.517	-0.164	1	*ns.*	gamma-Glutamyltryptophan	0.171	Pimelic acid	0.329
Paired data - Sugars	0.175	0.563	-0.584	1	*ns.*	Xanthine	0.194	Glucose	0.275
Paired data - Sucrose	0.179	0.592	-0.269	1	*ns.*	Methylmalonylcarnitine	0.209	Ribitol	0.391
Paired data - Starch	0.207	0.489	-0.0235	1	*ns.*	Valine	-0.160	LysoPC(0:0/20:3)	0.431
Paired data - Alcohol	0.176	0.566	-0.202	1	*ns.*	Taurodeoxycholic acid	-0.209	Lithocholic acid glycine conjugate	-0.376
Paired data - Monosaccharides	0.186	0.621	-0.00088	1	*ns.*	Butyrylcarnitine	0.153	4-Pyridoxic acid	0.582
Paired data - Disaccharides	0.177	0.59	-0.244	1	*ns.*	Progesterone	-0.171	4-Hydroxybenzoic acid	0.285
Paired data - Saturated fat	0.172	0.519	-0.478	1	*ns.*	2-Hydroxycaproic acid	-0.172	Isoleucyl-Leucine	0.284
Paired data - Monounsaturated fat	0.178	0.561	-0.327	1	*ns.*	LysoPC(20:0/0:0)	0.197	LysoPC(20:0/0:0)	0.375
Paired data - Polyunsaturated fat	0.096	0.626	-0.0851	1	*ns.*	LysoPE(20:4(5Z,8Z,11Z,14Z)/0:0)	-0.189	LysoPE(20:4(5Z,8Z,11Z,14Z)/0:0)	-0.313
Paired data - Total IBS-SSS	0.143	0.568	0.0564	0.293	*ns.*	Progesterone	-0.204	Sinigrin	0.401
Paired data - Abdominal pain	0.144	0.524	-0.127	1	*ns.*	Progesterone	-0.174	Sinigrin	0.459
Paired data - Diarrhea	0.139	0.59	-0.118	1	*ns.*	4-Hydroxybenzoic acid	0.199	4-Hydroxybenzoic acid	0.510
Paired data - Constipation	0.202	0.438	-0.0369	1	*ns.*	Valine	-0.148	Urea	-0.351
Paired data - Bloating and flatulence	0.145	0.564	0.00921	0.938	*ns.*	delta-Hexanolactone	-0.191	delta-Hexanolactone	-0.370
Paired data - Vomiting and nausea	0.185	0.393	-0.432	1	*ns.*	Propionylcarnitine	0.217	beta-Alanine	0.333
Paired data - Psychological well-being	0.154	0.617	-0.103	1	*ns.*	Ibuprofen	0.230	Ibuprofen	0.741
Paired data - Intestinal symptoms influence on daily life	0.185	0.618	0.0658	0.207	*ns.*	delta-Hexanolactone	-0.174	Sinigrin	0.487

Supplementary Table 2. Statistically significant metabolic features linking the Metaboanalyst pathway enrichment and Small Molecule Pathway Database (SMPDB) output with the Human Metabolome Database (HMDB) descriptions [26,27,39]. In total, 105 unique metabolic features were found significantly different between the intervention group and the control group, for the 4-week data and/or the paired data.

Annotated Metabolic features	Analysis	HMDB ID	KEGG ID	Baseline (T0)	Four weeks (T1)	Paired (1+(T1-T0)/T0)	MetaboAnalyst SMPDB pathway association	Pathway	HMDB description nutrient	HMDB description food sources
Dodecanoylcarnitine	LCMS (+)	HMDB0002250		*	***	***				
Linoleic acid	LCMS (-), GCMS	HMDB0000673	C01595	ns	***	***	Alpha Linolenic Acid and Linoleic Acid Metabolism	SMP00018	omega-6 fatty acid, PUFA essential	plant glycosides
Docosapentaenoic acid (22n-3)	LCMS (-)	HMDB0006528	C16513	ns	***	***	Alpha Linolenic Acid and Linoleic Acid Metabolism	SMP00018	omega-3 fatty acid essential	fish oils
Docosahexaenoic acid	GCMS, LCMS (-)	HMDB0002183	C06429	ns	***	***	Alpha Linolenic Acid and Linoleic Acid Metabolism	SMP00018	omega-3 fatty acid	fish oils
Palmitic acid	LCMS (-), GCMS	HMDB0000220	C00249	ns	***	***	Fatty Acid Biosynthesis	SMP00456	saturated fatty acid	palm fruit
3-Hydroxybutyric acid	LCMS (-), GCMS	HMDB0000011	C01089	ns	***	***	Fatty Acid Biosynthesis	SMP00456	ketone body	
(R)-3-Hydroxy-hexadecanoic acid	LCMS (-)	HMDB0010734		ns	***	***	Fatty Acid Biosynthesis	SMP00456	saturated fatty acid	
trans-2-Dodecenoylcarnitine	LCMS (+)	HMDB0013326		ns	***	***				cow milk
Palmitoleic acid	GCMS, LCMS (-)	HMDB0003229	C08362	ns	***	***			monounsaturated fatty acid	macadamia oil, sea buckthorn oil

Oleic acid	LCMS (-)	HMDB0000207	C00712	ns	***	***			monounsaturated fatty acid	olive oil, almond oil
Myristoleoylcarnitine	LCMS (+)	HMDB0240588		ns	***	***				
Myristoleic acid	LCMS (-)	HMDB0002000	C08322	ns	***	***			monounsaturated fatty acid	cow milk, sunflowers, dates
Octadecenoic acid,-9-(z)-	GCMS	HMDB0000573	C01712	ns	***	***			monounsaturated fatty acid	hydrogenated vegetable oils, milk
Eicoseneoylcarnitine	LCMS (+)			ns	***	***				
alpha-Dimorphecolic acid	LCMS (-)	HMDB0004670	C14767	ns	***	***			endogenous fatty acid	
3-Hydroxydodecanoic acid	LCMS (-)	HMDB0000387		ns	***	***			saturated fatty acid	
3-Hydroxycapric acid	LCMS (-)	HMDB0002203		ns	***	***			saturated fatty acid	
3, 5-Tetradecadiencarnitine	LCMS (+)	HMDB0013331		ns	***	***				
2-Hydroxymyristoylcarnitine	LCMS (+)	HMDB0013166		ns	***	***				
2-Hydroxymyristic acid	LCMS (-)	HMDB0002261		ns	***	***				
2-Hydroxylauroylcarnitine	LCMS (+)	HMDB0013164		ns	***	***				
Aconitic acid, cis-	GCMS	HMDB0000072	C00417	ns	**	***	Citric Acid Cycle	SMP00057		

Hexose	GCMS	HMDB0000122	C00221	ns	**	***	Transfer of Acetyl Groups into Mitochondria	SMP00466		
Oleoylcarnitine	LCMS (+)	HMDB0005065		ns	**	***				
Citric acid	GCMS, LCMS (-)	HMDB0000094	C00158	ns	**	***	Transfer of Acetyl Groups into Mitochondria	SMP00466		citrus fruits
2-amino-adipic acid	GCMS	HMDB0000510	C00956	ns	**	***				
L-Acetylcarnitine	LCMS (+)	HMDB0000201	C02571	ns	*	***	Beta Oxidation of Very Long Chain Fatty Acids	SMP00052		
Urea	GCMS	HMDB0000294	C00086	ns	*	***	Urea Cycle	SMP00059		
Decanoylcarnitine	LCMS (+)	HMDB0000651		ns	*	***				
9-Hexadecenoylcarnitine	LCMS (+)	HMDB0013207		ns	*	***				
9,12-Hexadecadienoylcarnitine	LCMS (+)	HMDB0013334		ns	*	***				
1,5-anhydro-d-glucitol	GCMS	HMDB0002712	C07326	ns	*	***				derived mainly from food
Octadecanoic acid	GCMS	HMDB0000827	C01530	ns	*	***	Mitochondrial Beta-Oxidation of Long Chain Saturated FA's	SMP00482	saturated fatty acid	animal and vegetable fats and oils
L-Octanoylcarnitine	LCMS (+)	HMDB0000791	C02838	ns	ns	***	Mitochondrial Beta-Oxidation of Short Chain Saturated FA's	SMP00482		

Tiglylcarnitine	LCMS (+)	HMDB0002366		ns	ns	***				
Hexadecanedioic acid	LCMS (-)	HMDB0000672	C19615	ns	ns	***			saturated fatty acid	
5,8,11-Eicosatrienoic acid	LCMS (-)	HMDB0010378		ns	ns	***			omega-9 fatty acid, PUFA	
3-Methylglutarylcarnitine	LCMS (+)	HMDB0000552		ns	ns	***				
N-gamma-Glutamylglutamine	LCMS (+)	HMDB0029147		ns	ns	***				
Dodecanoic acid	LCMS (-), GCMS	HMDB0000638	C02679	ns	ns	***	Beta Oxidation of Very Long Chain Fatty Acids	SMP00052	saturated fatty acid	coconut oil, palm kernel oil
Sebacic acid	LCMS (-)	HMDB0000792	C08277	ns	ns	***			saturated fatty acid	
Pimelic acid	LCMS (-)	HMDB0000857	C02656	ns	ns	***			saturated fatty acid	
Isovalerylcarnitine	LCMS (+)	HMDB0000688		ns	ns	***				
Isobutyryl-L-carnitine	LCMS (+)	HMDB0000736		ns	ns	***				
Dodecanedioic acid	LCMS (-)	HMDB0000623	C02678	ns	ns	***			saturated fatty acid	
delta-Hexanolactone	LCMS (-)	HMDB0000453		ns	ns	***				
3-Hydroxyisovalerylcarnitine	LCMS (+)	HMDB0061189		ns	ns	***				

Progesterone	LCMS (+)	HMDB0001830	C00410	*	***	**			steroid hormone	
LysoPC(20:3(8Z,11Z,14Z)/0:0)	LCMS (+)	HMDB0010394	C04230	ns	***	**				
LysoPC(0:0/20:3)	LCMS (+)		C04230	ns	***	**	Glycerophospholipid metabolism	SMP00039		
Valerylcarnitine	LCMS (+)	HMDB0013128		ns	**	**				cow milk
Hydroxyoctanoic acid	LCMS (-)	HMDB0000711		ns	**	**				
Stearoylcarnitine	LCMS (+)	HMDB0000848		ns	**	**	Mitochondrial Beta-Oxidation of Long Chain Saturated FA's	SMP00482		
Arachidonic acid	LCMS (-), GCMS	HMDB0001043	C00219	ns	**	**	Alpha Linolenic Acid and Linoleic Acid Metabolism	SMP00018	essential fatty acid, PUFA	
12-Hydroxystearic acid	LCMS (-)	HMDB0061706		ns	*	**				edible vegetable oils (canola oil, castor oil)
gamma-Glutamylalanine	LCMS (+)	HMDB0006248	C03740	ns	*	**	Glutathione Metabolism	SMP00015		
Cytidine	LCMS (-)	HMDB0000089	C00475	ns	*	**	Pyrimidine Metabolism	SMP00046		
Tetradecanoylcarnitine	LCMS (+)	HMDB0005066		ns	*	**				
N2,N2-Dimethylguanosine	LCMS (+)	HMDB0004824		ns	*	**				

2-Octenoylcarnitine	LCMS (+)	HMDB0013324		ns	ns	**				
cis-4-Decenoylcarnitine	LCMS (+)	HMDB0240585		ns	ns	**				
Malic acid	LCMS (-), GCMS	HMDB0000156	C00149	ns	ns	**	Transfer of Acetyl Groups into Mitochondria	SMP00466		apples, grapes
Hexanoylcarnitine	LCMS (+)	HMDB0000756		ns	ns	**				
Sinigrin	LCMS (-)	HMDB0034070	C08427	ns	ns	**				black mustard, cabbage, Brussels sprouts
LysoPC(20:4(5Z,8Z,11Z,14Z)/0:0)	LCMS (+)	HMDB0010395	C04230	ns	ns	**				
Myristic acid	LCMS (-), GCMS	HMDB0000806	C06424	ns	***	*	Fatty Acid Biosynthesis	SMP00456	saturated fatty acid	animal and vegetable fats
Acetaminophen	LCMS (+), GCMS	HMDB0001859	C06804	ns	**	*				drug, painkiller
LysoPC(14:0/0:0)	LCMS (+)	HMDB0010379	C04230	ns	**	*	Arachidonic Acid Metabolism	SMP00075		
Valine	GCMS	HMDB0000883	C00183	ns	**	*			essential amino acids	
LysoPC(0:0/14:0)	LCMS (+)		C04230	ns	**	*	Glycerophospholipid metabolism	SMP00039		
Pyroglutamylvaline	LCMS (+)	HMDB0094651		ns	*	*				

Glycine	GCMS	HMDB0000123	C00037	ns	*	*	Glutathione Metabolism	SMP00015	non-essential amino acid	
alpha-Linolenic acid	LCMS (-)	HMDB0001388	C06427	ns	*	*	Alpha Linolenic Acid and Linoleic Acid Metabolism	SMP00018	essential omega-3 fatty acid	vegetable oils, nuts
L-Palmitoylcarnitine	LCMS (+)	HMDB0000222	C02990	ns	*	*	Fatty acid Metabolism	SMP00051		
LysoPC(20:2(11Z,14Z)/0:0)	LCMS (+)	HMDB0010392	C04230	ns	*	*				
LysoPC(0:0/16:1)	LCMS (+)		C04230	ns	*	*	Glycerophospholipid metabolism	SMP00039		
LysoPC(22:6(4Z,7Z,10Z,13Z,16Z,19Z)/0:0)	LCMS (+)	HMDB0010404	C04230	ns	ns	*				
Butyrylcarnitine	LCMS (+)	HMDB0002013	C02862	ns	ns	*				
Pyroglutamic acid	GCMS	HMDB0000267	C01879	ns	ns	*	Glutathione Metabolism	SMP00015		urinebiomarker for cheese consumption
Cervonylcarnitine	LCMS (+)	HMDB0006510		ns	ns	*				
Eicosapentaenoic acid	LCMS (-)	HMDB0001999	C06428	ns	ns	*	Alpha Linolenic Acid and Linoleic Acid Metabolism	SMP00018	important PUFA	fish oils
Abietic acid	LCMS (-)			ns	ns	*				
3-Hydroxyisovaleric acid	LCMS (-)	HMDB0000754		ns	ns	*				prolonged consumption

										of raw egg-whites
L-Glutamine	LCMS (+)	HMDB0000641	C00064	ns	ns	*	Urea Cycle	SMP00059	non-essential amino acid	fish, red meat, beans, dairy products
2-Oxovaleric acid	LCMS (-)	HMDB0001865	C06255	ns	ns	*				
Uracil	LCMS (+)	HMDB0000300	C00106	ns	ns	*	Pyrimidine Metabolism	SMP00046		
Succinylcarnitine	LCMS (+)	HMDB0061717		ns	ns	*				
Hippuric acid	LCMS (-)	HMDB0000714	C01586	ns	ns	*				tea, wine, fruit juices
Inositol, scyllo-	GCMS	HMDB0006088	C06153	ns	ns	*				coconut palm
3-Hydroxybutyrylcarnitine	LCMS (+)	HMDB0013127		ns	ns	*				
Caprylic acid	LCMS (-)	HMDB0000482	C06423	**	***	ns	Beta Oxidation of Very Long Chain Fatty Acids	SMP00052	saturated fatty acid	coconuts
Adipic acid	GCMS	HMDB0000448	C06104	*	**	ns				additive and gelling agent in jello or gelatins
Chenodeoxycholic acid glycine conjugate	LCMS (-)	HMDB0000637	C05466	ns	**	ns	Bile Acid Biosynthesis	SMP00035		

Inosine	LCMS (-)	HMDB0000195	C00294	ns	**	ns	Purine Metabolism	SMP00050	
Eicosanedioic acid	LCMS (-)			ns	**	ns			
Glycoursodeoxycholic acid	LCMS (-)	HMDB0000708		ns	*	ns			
Sucrose	GCMS	HMDB0000258	C00089	ns	*	ns	Starch and Sucrose Metabolism	SMP00058	sugarcane, sugar beet
Eicosadieneoylcarnitine	LCMS (+)			ns	*	ns			
Linoleylcarnitine	LCMS (+)	HMDB0006469		ns	*	ns			
LysoPC(18:3(9Z,12Z,15Z)/0:0)	LCMS (+)	HMDB0010388	C04230	ns	*	ns			
Theobromine	LCMS (+)	HMDB0002825	C07480	ns	*	ns			cacao bean
Guanosine	LCMS (+)	HMDB0000133	C00387	ns	*	ns	Purine Metabolism	SMP00050	leeks, garlic, black-eyed peas
Fructose	GCMS	HMDB0000660	C02336	ns	*	ns	Starch and Sucrose Metabolism	SMP00058	honey, tree fruits, berries
LysoPC(16:1(9Z)/0:0)	LCMS (+)	HMDB0010383	C04230	ns	*	ns			
LysoPC(0:0/15:0)	LCMS (+)		C04230	ns	*	ns	Glycerophospholipid metabolism	SMP00039	

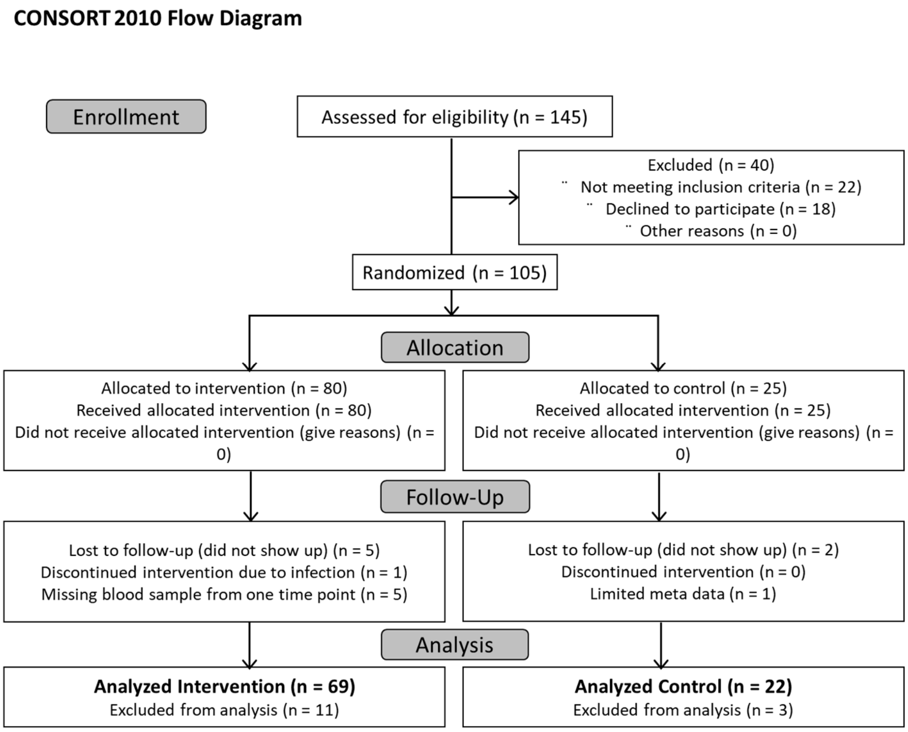

Supplementary Figure 2. CONSORT 2010 Flow Diagram.

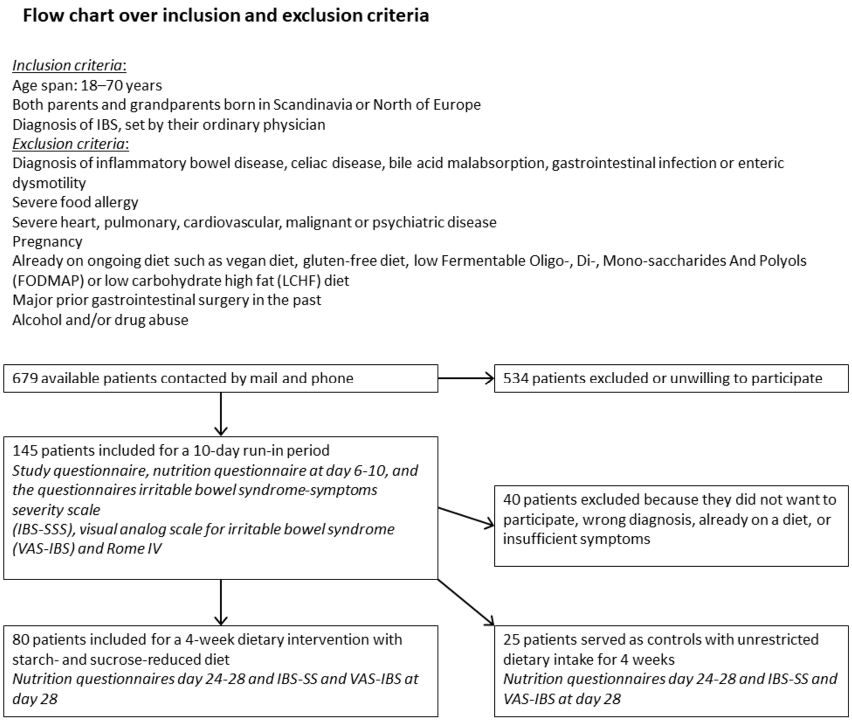

Supplementary Figure 3. Flow chart over inclusion and exclusion criteria.

Supplementary Table 3. Recommendations for berries, fruit, legumes, and vegetable intake according to starch- and sucrose-reduced diet (SSRD). The dietary recommendations were downloaded from www.sucroseintolerance.com/choosing-your-foods/ [22]. *excess intake can cause bloating/flatulence in all individuals.

Berries and fruits

Well tolerated	Tolerated by some	Not tolerated
Avocado	Persimmons	Apples
Blackberries	Plums	Apricots
Blueberries	Raisins	Bananas
Boysenberries	Watermelon	Cantaloupe
Cherries		Dates
Cranberries		Grapefruits
Currants		Guava
Figs		Honeydew melon
Gooseberries		Mangos
Grapes		Nectarines
Kiwi fruits		Oranges
Lemons		Passion fruits
Limes		Peaches
Loganberries		Pineapples
Olives		Tangelos
Papayas		Tangerines
Pears		
Pomegranates		
Prunes		
Raspberries		
Rhubarbs		
Strawberries		

Vegetables and legumes

Well tolerated	Tolerated by some	Not tolerated
Alfafa sprouts	Edamame soybeans	Beets
Artichokes*	Jicamas	Black beans
Arugulas	Leeks	Black-eyed peas
Asparagus*	Okra	Butternut
Bamboo shoots	Pumpkins	Carrots
Bok choy	Snow peas	Cassavas
Broccoli*	Tempeh	Chickpeas
Brussels sprouts*	Tofu	Corn
Cabbages*	Yellow wax beans	Garlic
Cauliflower*		Green peas

Celery	Lentils
Chard	Kidney beans
Chicories	Lima beans
Chives	Navy beans
Collard greens	Onion
Cress	Parsnips
Cucumbers	Pinto beans
Eggplants	Potatoes
Endive	Soybeans
Green beans	Split peas
Kale	Sweet potatoes
Lettuces	Yams
Mung bean sprout	
Mushrooms	
Mustard green	
Peppers	
Radishes	
Spaghetti squash	
Spinach	
Tomatoes	
Turnips	
Yellow squash	
Zucchini	
